# Perception de la gravité de la maladie cancéreuse et pluralisme thérapeutique en oncologie médicale à l’Hôpital Général de Yaoundé

**DOI:** 10.11604/pamj.2023.44.96.27765

**Published:** 2023-02-17

**Authors:** Zacharie Sando, Anne Christiane Essama Nnomo, Lionel Tabola, Julienne Ngo Likeng, Grace Nganwa, Etienne Atenguena, Berthe Sabine Esson Mapoko, Anne Sango, Paul Ndom, Sylvanie Makou Njombou, Linda Sando Ngueffo, Benjamin Alexandre Nkoum

**Affiliations:** 1 Faculté de Médecine et des Sciences Biomédicales, Université de Yaoundé I, Yaoundé, Cameroun,; 2 Ecole des Sciences de la Santé, Université Catholique d´Afrique Centrale, Yaoundé, Cameroun,; 3 Faculté de Médecine et des Sciences Pharmaceutiques, Université de Douala, Douala, Cameroun,; 4 University of Buea, Buea, Cameroon,; 5 Women Impetus for a Development (WIFAD), Cameroun

**Keywords:** Oncologie, adultes, perception, gravité, pluralisme thérapeutique, Hôpital Général de Yaoundé, Oncology, adults, perception, severity, therapeutic pluralism, General Hospital of Yaoundé

## Abstract

**Introduction:**

le cancer est une pathologie potentiellement grave. L´annonce d´une maladie comme le cancer est une mauvaise nouvelle. Le vécu de cette annonce est différent en fonction de chaque personne. Le comportement des proches a une influence sur le comportement du patient ainsi que sur l´observance thérapeutique. L´utilisation de traitements alternatifs est une pratique courante en oncologie dans certains pays africains. L´objectif était d´établir le ressenti des patients face au cancer, l´ampleur de l´utilisation des traitements alternatifs, ainsi que des facteurs influençant leurs choix.

**Méthodes:**

de décembre 2019 à mai 2020, nous avons mené une étude descriptive à l´Hôpital Général de Yaoundé. Ont été inclus dans cette étude les patients de plus de 18 ans pris en charge pour pathologie cancéreuse, ayant débuté la chimiothérapie depuis au moins trois mois, et qui ont accepté de répondre au questionnaire.

**Résultats:**

cent vingt-deux (122) patients ont été interrogés. Le sexe ratio était de 1/1. La moyenne d´âge était de 45 ans. Parmi les participants, 38,5% pensaient que le cancer est une maladie très grave, 24% des patients étaient désespérés à l´annonce du diagnostic, 61% pensaient que la guérison devait être très lente. Les pluralistes de notre échantillon représentaient 59,8%.

**Conclusion:**

les patients porteurs d´un cancer ainsi que leurs proches le perçoivent généralement comme grave. A l´annonce du diagnostic, les malades sont généralement paniqués. Le pluralisme thérapeutique est une pratique fréquente.

## Abstract (en)

**Introduction:**

cancer is a potentially serious disease. The announcement of a cancer diagnosis is bad news. This diagnosis is experienced differently from one person to the next. Patient's behaviour and compliance reflects specific behaviors of relatives. Alternative treatments are commonly used in oncology in some African countries. The purpose of this study was to establish cancer patients' experience, the extent of the use of alternative treatments and factors influencing their choices.

**Methods:**

we conducted a descriptive study at the Yaoundé General Hospital from December 2019 to May 2020. The study included patients over 18 years of age treated for cancer, who had been undergoing chemotherapy for at least three months and who agreed to complete the questionnaire.

**Results:**

the interview involved 122 patients. Sex ratio was 1/1. The average age of patients was 45 years; 38.5% of patients thought that cancer is a very serious disease, 24% were desperate for diagnosis, 61% thought that recovery would be very slow. Pluralists in our sample accounted for 59.8%.

**Conclusion:**

cancer patients and their relatives generally perceive cancer as serious. Patients experience a feeling of sudden and intense anxiety when they are diagnosed with cancer. Therapeutic pluralism is a frequent practice.

## Introduction

Le cancer est une pathologie potentiellement grave. En 2018, on comptait près de 18 millions de nouveaux cas et 9,5 millions de décès liés à cette maladie [1]. Plus de 60% des nouveaux cas de cancer surviennent en Afrique, en Asie, en Amérique centrale et en Amérique latine. Ces régions représentent 70% des décès par cancer dans le monde [1]. L´annonce d´une maladie grave comme le cancer est une mauvaise nouvelle, il s´agit donc d´une information qui modifie radicalement et négativement l´idée que se fait le patient de son avenir [2]. Le vécu de cette annonce est différent en fonction de chaque personne. Ce vécu conditionne la compréhension de la maladie et l´adhésion thérapeutique, en fonction du contexte socio-économique et familial, de la gravité ressentie de la maladie mais aussi de la représentation qu´elle se fait de la maladie. La gestion de leur maladie par les patients et leur adaptation sont liées à la façon dont leur réseau social, en particulier leur famille, voit cette maladie [3]. Le comportement des proches a une influence sur le comportement du patient, l´expression des symptômes, et même, le cours réel de la maladie, dans ses composantes d´évolution symptomatique, ainsi que sur l´observance thérapeutique [4]. La médecine allopathique propose des traitements ayant faits leurs preuves. Pourtant, l´utilisation de traitements alternatifs est une pratique courante en oncologie pédiatrique dans certains pays africains [5]. La médecine alternative est toute intervention médicale n´étant pas généralement enseignée à la faculté de médecine et n´étant pas disponible dans les arrangements habituels de soin [6]. Ces traitements sont dans la plupart utilisés par les patients avec pour but de traiter le cancer [5]. Au Cameroun, il existe peu de données publiées sur le regard porté par les malades sur la gravité du cancer, ainsi que les traitements alternatifs en oncologie médicale. Raison pour laquelle, nous avons réalisé cette étude. Elle vise à établir le ressenti des patients face au cancer, l´ampleur de l´utilisation des traitements alternatifs, ainsi que des facteurs influençant leurs choix.

## Méthodes

**Type d´étude:** nous avons mené une étude descriptive à l´Hôpital Général de Yaoundé.

**Lieu et population d´étude:** l´Hôpital Général de Yaoundé est l´Hôpital de référence de la prise en charge des cancers dans la ville de Yaoundé. Yaoundé est la capitale du Cameroun, pays de l´Afrique Centrale. L´étude a été réalisée de décembre 2019 à mai 2020. La population d´étude était constituée des patients atteints d´un cancer. Nous avons interrogé les patients en hospitalisation conventionnelle, en hospitalisation du jour, ou en consultation externe. Ont été inclus dans cette étude les patients de plus de 18 ans, ayant un diagnostic confirmé de cancer, pris en charge depuis au moins trois mois, s´exprimant en français ou en anglais, et qui ont accepté de répondre au questionnaire.

**Variables:** le patient s´exprimait sur ce qu´il pensait (gravité de la maladie, célérité de la guérison), ressentait (à l´annonce du diagnostic) ou encore sur ce que ses proches lui avaient fait penser. En outre, on a répertorié les traitements alternatifs à son traitement conventionnel qu´il consommait. Le traitement alternatif était tout traitement utilisé par les patients, mais qui n´avait pas été prescrit en milieu hospitalier. Les patients utilisant au moins un traitement alternatif étaient considérés comme pluralistes. Le pluralisme modéré correspondait à l´utilisation d´un ou deux traitements alternatifs. Le pluralisme exagéré correspondait à l´utilisation d´au moins trois traitements alternatifs différents. Nous avons également demandé la durée pendant laquelle le traitement alternatif a été utilisé.

**Outil de collecte de données:** un questionnaire pré établi comportant 15 questions a permis de recueillir les différentes données. Il a été pré testé sur une dizaine de patients.

**Collecte des données:** les données démographiques telles que l’âge, le sexe, le niveau d’étude ont été collectées. Le questionnaire a été administré par le même investigateur.

**Taille de l´échantillon:** notre échantillonnage a été consécutif non probabiliste.

**Analyse des données:** l´analyse statistique a été réalisée grâce au logiciel Epi info 5. L´analyse descriptive a été faite, avec recueil des effectifs et pourcentage. Pour les variables quantitatives comme l´âge, nous avons calculé médiane et déviations standard.

**Considérations éthiques:** nous avons obtenu une autorisation de recherche auprès de l´administration de l´Hôpital Général de Yaoundé. Le consentement libre et éclairé de chaque patient était obtenu avant sa participation.

## Résultats

**Caractéristiques sociodémographiques:** nous avons enquêté auprès de 122 patients. La moyenne d´âge était de 45 ans avec des extrêmes de 18 et 86 ans. Le sexe ratio était de 1/1. Parmi les patients, 65% avaient un niveau secondaire, et 2,5% n´avaient jamais été scolarisés (Tableau 1).

**Tableau 1:** caractéristiques sociodémographiques des patients, Yaoundé, 2020 (N = 122)

Items	Effectif N=122	Pourcentage (%)
**Sexe**		
Masculin	61	50
Féminin	61	50
**Age**		
< 20 ans	1	0,8
20-39 ans	65	53,2
40-59 ans	39	32
60-79 ans	14	11,5
≥ 80 ans	3	2,5
**Niveau d’instruction**		
Aucun	3	2,5
Primaire	22	18
Secondaire	65	53,3
Supérieur	32	26,2

**Perception de la gravité de la maladie par le patient:** parmi les participants, 38,5% pensaient que le cancer est une maladie très grave, et 9% pensaient que le cancer n´est pas grave. A l´annonce du diagnostic, 24% des patients étaient désespérés. Quant à la célérité de la guérison, 61% pensaient que la guérison devait être très lente (Tableau 2).

**Tableau 2:** perception de la gravité de la maladie par le patient, Yaoundé, 2020 (N = 122)

Items	Effectif N =122	Pourcentage (%)
**Connaissance théorique du cancer avant le diagnostic**		
Oui	120	98.4
Non	2	1.6
**Avis sur la présence du cancer dans l’organisme longtemps avant le diagnostic**		
Oui	79	64.8
Non	43	35.2
**Avis sur la gravité du cancer au moment du diagnostic**		
Pas grave	11	9
Grave	64	52.5
Très grave	47	38.5
**Opinion sur la célérité de guérison**		
Guérison très lente	61	50
Guérison lente	25	20.5
Guérison rapide	36	29.5
**Réaction à l’annonce du diagnostic**		
Désespoir	24	19.7
Tristesse / traumatisme	5	4.1
Peur / panique	66	54.1
Calme	10	8.2
Optimisme / Confiance	17	13.9

**Perception de la gravité de la maladie par les proches:** chez 70,5% des patients, plus d´une personne de leur entourage leur avait fait penser que leur maladie était grave. Elle était jugée incurable par au moins un proche de 54,1% des patients (Tableau 3).

**Tableau 3:** perception de la gravité de la maladie par les proches, Yaoundé, 2020 (N = 122)

Items	Effectif N =122	Pourcentage (%)
**Nombre de proches ayant fait penser au patient que le cancer est grave**		
Aucune personne	36	29.5
Une personne	36	29.5
Plus d’une personne	50	41
**Pensée par au moins un proche que le cancer est incurable**		
Oui	66	54.1
Non	56	45.9

### Profil thérapeutique

**Pluralisme thérapeutique:** dans notre population, 59,8% des enquêtés se livraient au pluralisme thérapeutique (Tableau 4).

**Tableau 4:** Spluralisme thérapeutique, Yaoundé, 2020 (N=122)

Items	Effectif (N=122)	Pourcentage (%)
Pluralisme inexistant	49	40,2
Pluralisme modéré	39	32
Pluralisme exagéré	34	27,8

**Profil des utilisateurs de traitement alternatif:** parmi les patients utilisant les traitements alternatifs, 60,3% pensaient qu´ils accélèrent la guérison. L´utilisation de traitement alternatif était sur initiative personnelle dans 49,3% des cas et sur proposition des proches dans 76,7%. Les traitements alternatifs étaient utilisés depuis plus d´un mois par 74% des pluralistes (Tableau 5).

**Tableau 5:** profil des utilisateurs des traitements alternatifs (pluralistes), Yaoundé, 2020 (N = 73)

Items	Effectif N = 73	Pourcentage (%)
**Pensée que les traitements alternatifs accélèrent la guérison**		
Oui	44	60,3
Non	29	39,7
**Prise de traitement alternatif sur initiative personnelle**		
Oui	36	49,3
Non	37	50,7
**Prise de traitement alternatif proposé par l’entourage**		
Oui	49	67,1
Non	24	32,9
**Evolution de l’état général durant la prise de traitement alternatif**		
Amélioration	1	1,4
Stationnaire	46	63
Dégradation	26	35,6
**Durée d’utilisation de traitement alternatif**		
< 30 jours	19	26
≥ 30 jours	54	74
**Temps écoulé depuis l’annonce du diagnostic**		
< 6 mois	17	23,3
6 mois - 12 mois	29	39,7
> 12 mois	27	37

## Discussion

### Perception de la gravité de la maladie par le patient

#### Vécu de l´annonce de la maladie par le patient

Dans notre étude, les réactions des patients au moment de l´annonce étaient différentes les unes des autres. Parmi ces réactions, ont été identifiés: le désespoir, la tristesse, le traumatisme, la peur, la panique, le calme, la confiance. Les réactions du patient face aux mauvaises nouvelles retrouvées dans la littérature sont : l´incrédulité (« ce n´est pas vrai »), le choc, la dénégation, le déplacement, la peur et l´anxiété, la colère et les reproches, la culpabilité, l´espoir, le désespoir et dépression, le soulagement, l´humour [2]. Ces réactions peuvent être réparties en trois niveaux: des réactions psychophysiologiques telles que l´agitation, une activité psychomotrice accrue, un effondrement avec retrait et mutisme; des réactions cognitives, telles que le déni, le blâme, l´intellectualisation, l´incrédulité, l´acceptation ; et des réactions affectives, telles que la colère, la peur, l´anxiété, l´impuissance, le désespoir, la honte, la culpabilité, la délivrance [7].

### Perception de la gravité de la maladie cancéreuse par le malade

La quasi-totalité (91%) des patients pensaient que le cancer est une pathologie grave, voire très grave. L´annonce du diagnostic d´un cancer est ressentie le plus souvent comme l´entrée dans la maladie grave [8]. L´Organisation mondiale de la Santé définit la santé comme un état complet de bien-être physique, mental et social et ne consiste pas seulement en une absence de maladie ou d´infirmité. Il n´existe pas de définition précise de ce qu´est une maladie grave, la notion de gravité étant subjective. La crainte d´une maladie est liée au risque de souffrance et de mort. Le cancer suscite de fortes appréhensions, car elle est souvent vécue comme une perte de contrôle de son propre corps mais aussi parce qu´elle nous confronte à la mort. Cette mort qui sans être abordée directement est pourtant omniprésente durant l´entretien d´annonce de la maladie [9]. La représentation du cancer s´ancre dans l´Histoire où « pendant bien longtemps en Occident et aujourd´hui encore dans certaines sociétés traditionnelles », notamment africaines, « les pathologies mortelles étaient interprétées comme relevant d´une malédiction, d´un mauvais sort jeté, ou résultant d´un envoûtement » [10]. Le cancer est parfois perçu comme une maladie qui menace à la fois la vie et la féminité, qui entraine des problèmes physiques, psychologiques, sexuels et professionnels, qui a des périodes de récupération et d'exacerbation, et qui provoque des troubles de l'adaptation à court et long terme [11]. Le quart (26,2%) de notre population avait fait des études universitaires. Divers aspects sociodémographiques tels que le statut scolaire, l´âge, la profession sont des déterminants importants de la perception de la maladie cancéreuse [12]. Pour les patients avec un niveau d'éducation élevé le cancer a un impact émotionnel moindre [12].

### Perception de la gravité de la maladie cancéreuse par les proches

Dans notre étude, les proches ont une influence dans le vécu de l´annonce de la maladie. Pour eux, le cancer est grave et même incurable. Ce que vivent les membres de la famille peut exercer une influence sur l´expérience du malade. La maladie intervient comme une crise, elle fait irruption dans le système familial et rompt l´équilibre établi. Toute famille fonctionne comme un système régi par des lois qui lui sont propres et qui définissent la place de chacun, il faut alors trouver un nouvel équilibre qui dépend des capacités de la famille à s´adapter au changement. L´entourage a un rôle bienveillant indispensable pour soutenir le malade. Il conditionne le sentiment de sécurité et de stabilité affective sur lequel le malade peut prendre appui et puiser de la force [13]. On attend des proches d´être une source de soutien mais eux aussi sont bousculés par l´irruption de la maladie grave [14]. Les membres de la famille sont des compagnons importants et une source irremplaçable de soutien pour les patients souffrant de maladie grave [15,16]. Ils apportent un soutien émotionnel aux patients et jouent un rôle considérable chez les patients dans le choix d'un oncologue traitant, d'un hôpital et dans les décisions sur les options de traitement possibles. Cependant les interactions sociales négatives peuvent entraîner plus de détresse psychologique chez les patients atteints de cancer [17]. Toute maladie grave nécessite de prendre en compte un triangle de soin constitué par le patient, sa famille et les soignants.

### Pluralisme thérapeutique

Dans notre échantillon, 59,8% des enquêtés étaient pluralistes, ils utilisaient au moins un traitement alternatif. Ces résultats sont corroborés par plusieurs études montrant que plus de 50% des patients ayant un cancer ont recours à une ou plusieurs thérapies alternatives [5,18,19]. Ces traitements étaient consommés sur initiative personnelle dans la moitié des cas, et sur conseil des proches dans deux tiers des cas.

Les 3/5 des pluralistes pensaient que l´utilisation de traitements alternatifs accéléraient la guérison. Cette proportion se rapproche des résultats d´une étude américaine dans laquelle 4/5 des patients cancéreux pensaient qu´utiliser concomitamment le traitement conventionnel et le traitement alternatif était préférable à n´utiliser que le traitement conventionnel [20]. Une étude au Maroc, retrouvait la guérison comme étant la première raison d´utilisation des thérapies alternatives [5]. Lors de l´utilisation des traitements alternatifs, le tiers des malades a vu son état général se dégrader. Dans la littérature, une étude comparant deux groupes de patients cancéreux l´un n´utilisant que le traitement conventionnel et l´autre utilisant le traitement alternatif, a révélé que l´évolution est nettement plus favorable pour le groupe n´ayant pas eu recours à une thérapie alternative [21]. Les patients partisans des thérapies complémentaires, ont souvent choisi de suivre un traitement conventionnel incomplet, ce qui suffit sans doute à expliquer leurs moins bons résultats cliniques. L´emploi de telles thérapies est donc corrélé avec le refus d´une partie du traitement conventionnel par les patients et a pour conséquence une diminution sensible de leur survie. On devrait accorder une plus grande attention à l´emploi de traitements alternatifs de patients cancéreux, pour s´assurer de l´observance du traitement anti-tumoral par ces patients. La majorité des pluralistes utilisaient le traitement alternatif depuis plus d´un mois. Cette utilisation prolongée de ces traitements grèverait davantage le pronostic des patients. En effet, en Afrique sub-saharienne, la plupart des patients atteints d´un cancer arrive en consultation spécialisée d´oncologie étant déjà à un stade avancé de la maladie [22].

**Limites:** notre étude a été réalisée en milieu hospitalier, et dans un seul centre. La généralisation des résultats est délicate parce qu´une proportion de patients se retrouve dans la communauté, patients ayant des avis différents de ceux interrogés. Dans notre étude, les patients faisaient recours à leurs souvenirs, ce qui auraient pu biaiser certaines réponses.

## Conclusion

Le cancer est une maladie grave. Les patients qui en sont porteurs le perçoivent généralement comme tel. Leurs proches en ont également la même perception. A l´annonce du diagnostic, les malades sont généralement paniqués voire désespérés. Le pluralisme thérapeutique est une pratique fréquente, souvent motivé par une envie de guérison rapide. Les traitements alternatifs sont utilisés au long cours.

### Etat des connaissances sur le sujet

- L´annonce d´une maladie grave comme le cancer est une mauvaise nouvelle;
- Les patients traités pour cancer ont souvent recours à la médecine alternative.

### Contribution de notre étude à la connaissance

- Au Cameroun, les patients atteints de cancer pensent que c´est une pathologie grave, voire très grave;
- Au Cameroun, la majorité des patients atteints de cancer utilisent des traitements alternatifs pensant accélérer leur guérison.
